# The phenomenon of degeneration of industrial *Trichoderma reesei* strains

**DOI:** 10.1186/s13068-021-02043-4

**Published:** 2021-10-01

**Authors:** R. Martzy, T. M. Mello-de-Sousa, R. L. Mach, D. Yaver, A. R. Mach-Aigner

**Affiliations:** 1 Christian Doppler Laboratory for Optimized Expression of Carbohydrate-Active Enzymes, Institute of Chemical, Environmental and Bioscience Engineering, TU Wien, Gumpendorfer Str. 1a, 1060 Vienna, Austria; 2 Institute of Chemical, Environmental and Bioscience Engineering, TU Wien, Gumpendorfer Str. 1a, 1060 Vienna, Austria; 3 Production Strain Technology, Novozymes Inc., Davis, CA USA

**Keywords:** *Trichoderma reesei*, *Hypocreajecorina*, Filamentous fungi, Industry strains, Cellulases, Degeneration, Chromatin

## Abstract

**Background:**

Even if the loss of production capacity of a microorganism is said to be a serious problem in various biotechnology industries, reports in literature are rather rare. Strains of the genera *Trichoderma reesei* are used for large-scale production of cellulases, which are needed in food and feed, textile, paper industries and biofuel production.

**Results:**

Here, we describe the phenomenon of spontaneous degeneration of *T. reesei* strains during large-scale cultivation. The phenotype of the degenerated population is characterized most importantly by a loss of any cellulase formation. Interestingly, promoter regions of relevant genes had a more compact chromatin in the (cel −) strains compared to productive strains. For a systematic investigation of the phenomenon a protocol for artificially induced and lab-scaled strain degeneration was developed. This workflow allows to determine the degeneration rate and thus, to compare the occurrence of a degenerated population in differently productive strains on the one hand, and to monitor the success of any strategies to prevent or decrease the degeneration on the other hand. While highly productive strains have higher degeneration rates compared to moderate producers, the degeneration can hardly be triggered in moderate producers. The observed (cel −) phenotype is not caused by a mutation in the gene encoding the essential transactivator Xyr1. The development of a non-producing population is also not triggered by any compounds released by either producing or non-producing cells.

**Conclusions:**

The extent of the occurrence of a degenerated strain population relates to the production capacity of the strain and goes along with chromatin condensation in relevant promoter regions.

## Background

In nature, *Trichoderma reesei* is as a saprotrophic fungus an excellent producer of enzymes for break-down of plant biomass. In industry, these carbohydrate-active enzymes are used for a number of applications: for example, xylanases are used in the food industry as a baking agent and for clarification of juice and wine [1] or in the paper industry for de-inking [2]. Cellulases from *T. reesei* are applied in the textile industry, for example for fibre polishing [3] or in the paper industry for recycling processes [4]. For the production of ethanol from cellulosic raw material (“cellulose ethanol”) cellulases obtained from *T. reesei* have a key role as well. In this process they are used to release D-glucose from the lignocellulosic biopolymers, which is subsequently used as carbon source in the sugar-to-ethanol fermentation (e.g., [5, 6] and citations therein).

Because of the broad application possibilities of *T. reesei* enzymes, and due to the outstanding secretion capacity of the fungus (around 100 g/L [7]), *T. reesei* is used for industry-scale production of some carbohydrate-active enzymes. Genome-wide analysis of this fungus identified 34 genes coding for cellulolytic and xylanolytic enzymes (reviewed in [8]), of which the most prominent cellulases are the cellobiohydrolases CBHI and CBHII (EC 3.2.1.91) [9] and the most studied xylanases are the endo-β-1,4-xylanases XYNI and XYNII (EC 3.2.1.8) [10]. The expression of the carbohydrate-active enzymes in *T. reesei* is regulated mainly at the transcriptional level in response to available carbon sources. Under glucose-limiting conditions the gene transcription is de-repressed; the full activation of cellulase production requires the presence of an inducing carbohydrate, e.g., cellulose or β-linked disaccharides such as cellobiose, sophorose, gentiobiose or lactose [11]. The full activation of xylanase expression is likewise dependent on the presence of xylan or xylan-derived carbohydrates such as D-xylose or xylobiose [11].

The main and indispensable transactivator of both regulons (cellulase- and xylanase-encoding genes) is the Xylanase regulator 1 (Xyr1) [12, 13]. Xyr1 is a binuclear Zn-cluster DNA-binding protein. It is generally expressed at a basal, rather low level; only by the cellulase expression-activating substance sophorose its own expression is also induced [14, 15]. Besides this, these regulons are either directly or indirectly subjected to carbon catabolite repression mainly mediated by the zinc finger protein Cre1. Cre1 does not only antagonize the transactivating function of Xyr1, it also represses *xyr1* expression itself. In this context, it is important to note that industry strains lack the full-length Cre1 protein [16, 17]. This is a property that industrial stains inherited since most of them were obtained by several mutagenesis steps from the *T. reesei* strain Rut-C30 [15, 18]. Rut-C30 is available for public research and was initially described as a hyper-cellulolytic strain [19, 20] and is itself a descendant of the *T. reesei* wild-type QM6a.

During industry-scale cellulase production using hyper-productive *T. reesei* strains it occurs that the microorganism spontaneously loses productivity during the fed-batch fermentation process. Certainly, this poses a threat to the biotechnological production process because this degeneration phenomenon is not understood nor can it currently be prevented. In this study we detailed investigated the phenomenon of strain degeneration, i.e., the arising (cel −) phenotype in *T. reesei* for the first time, and shed some light on possible regulatory mechanisms. Besides, we developed a lab-scale protocol for artificially induced strain degeneration that enables a standardized investigation of the extent of degeneration, i.e., the degeneration rate. This protocol will also allow monitoring the success of any future measures aiming to reverse or prevent the degeneration of strains.

## Results

### The phenomenon of degeneration of industrial strains

**Fig. 1:**
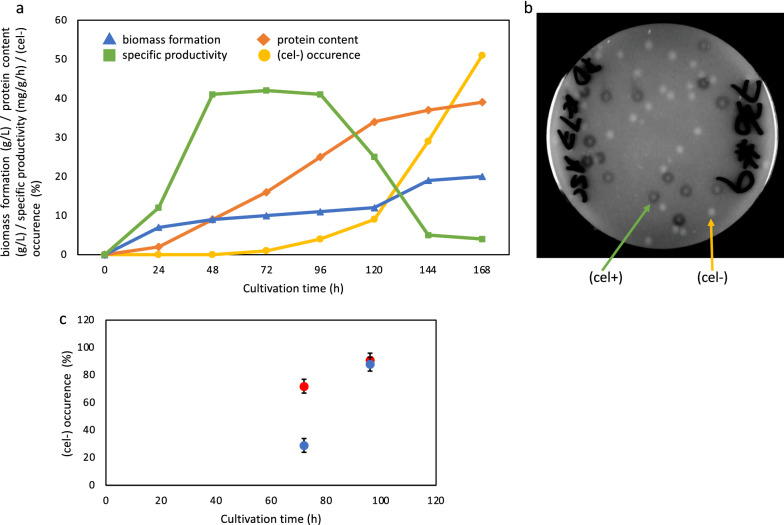
Occurrence of a (cel −) population during cellulase production. *T. reesei* Iogen-M10 strain was cultivated in a 14-L bioreactor under cellulase producing conditions. **a** Samples were taken every 24 h and used for determination of biomass formation (g/L; blue triangles), protein content (g/L; red diamonds), specific productivity (mg/g/h; green squares) and cellulase activity [% (cel −) population; yellow dots]. **b** For determination of cellulase activity the samples were spread on PDA plates for sporulation and then transferred to plates containing 1% (w/v) ASC and incubated for 144 h. The number of colonies without a halo, i.e., (cel −) colonies, and the number of colonies with a halo, i.e., (cel  +) colonies were counted. **c** Determination of the development of a (cel −) population in samples after 72 and 96 h of cultivation in the presence (blue) and absence of (red) of Trichostatin A. Values are means of three replicates, error bars indicate standard deviations

*T. reesei* Iogen-M10 is a highly productive strain concerning cellulase formation. However, it can unpredictably happen during the cultivation process for cellulase production that it loses its productivity. An example for the occurrence of such degeneration phenomenon is displayed in Fig. 1. After approx. 72 h of cultivation, the specific productivity starts to decrease and at the same time a non-producing population rises. In course of the remaining cultivation time this (cel −) population quickly becomes the dominant population and usually, the more abundant the (cel −) population becomes, the stronger the biomass formation increases (Fig. 1a, 144 and 168 h). The isolation of non-producing colonies and the transfer to fresh full or inducing medium does not restore their cellulase production ability; therefore, the phenomenon needs to be considered as irreversible. Besides this, (cel −) cells possess a different morphology: hyphal cells are less bulbous and more elongated. According to Biolog investigations, they also have a reduced ability to metabolize cellulase-inducing carbohydrates such as lactose, cellobiose as well as several other carbon compounds, such as xylose, ribose, or arabinose. A third characteristic is that the occurrence of a (cel −) population is unpredictable, which makes any investigation difficult. However, observations over years suggested that better producing strains seem to degenerate more likely than moderately producing strains.

### The development of a (cel −) population might be driven by epigenetic mechanisms

One obvious reason that could genetically cause the (cel −) phenotype would be a non-function mutation in the main transactivator Xyr1. However, a comparison of the genomic sequences of the *xyr1* structure gene (including a 900-bp-long upstream region from ATG) between Iogen-M10 and its (cel −) counterpart strain did not reveal any differences. On the other hand, when degeneration occurs, besides productive cells and (cel −) cells, a third type of cell was isolated from mid- to late-fermentation stages. These isolates form on acid-swollen cellulose (ASC) medium a smaller clearing zone compared to productive colonies, which form a large clearing zone, and a larger clearing zone compared to (cel −) colonies, which form no clearing zone at all (compare Fig. 1b). Serial passages of these (semi-cel −) cells to fresh ASC plates led to a slow loss of their ability to form any clearing zone, indicating the development to the full (cel −) phenotype. This gradual development from (semi-cel −) to (cel −) suggests that this phenotype development could be the result of epigenetic mechanisms. For this reason, biomass samples, which were taken after 72 h and 96 h of the above-mentioned cultivation for cellulase production, were divided and spread on PDA plates and PDA plates containing additionally Trichostatin A. These samples were chosen because according to the cellulase activity tests the (cel −) population starts to develop within this time period (compare Fig. 1a). Trichostatin A is an inhibitor of histone deacetylation and is expected to prevent formation of heterochromatin. After growth and sporulation of the fungal cells on the PDA plates with and without Trichostatin A, spores were harvested and spread on ASC plates for determination of cellulase activity. Interestingly, in the case of the earlier taken sample (72 h) the (cel −) appearance could be prevented to a strong extent by the presence of Trichostatin A, while the later taken biomass sample (96 h) exhibited the same high (cel −) population regardless whether it was treated with Trichostatin A (Fig. 1c). This is an indication that the development, however, not necessarily the initiation, of the (cel −) population might be driven by epigenetic mechanisms and could lead to higher heterochromatin formation in a (cel −) strain.

### The (cel −) phenotype is related to heterochromatin formation

**Fig. 2:**
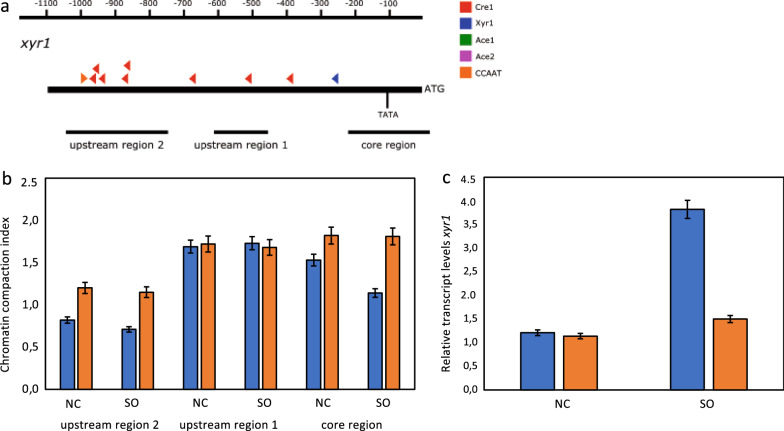
Analysis of the chromatin status upstream of the *xyr1* gene and its transcript formation. **a** Schematic drawing of the upstream region of the *xyr1* gene. The black bars indicate the regions chosen for the CHART-PCR. Triangles indicate putative binding sites for transcription factors (colour-code provided). TATA indicates the position of a TATA-like box. Numbers on top indicate distance from ATG. *T. reesei* Iogen-M10 (blue bars) and Iogen-M10 (cel −) (orange bars) strains were pre-grown and transferred to sophorose (SO) or medium without carbon source (NC) and incubated for 3 h. **b** CHART-PCR analysis of the three chosen regions. The chromatin compaction index (CCI) indicates the accessibility of DNA to DNase I digestion using normalization to *sar1* and *act* upstream regions. Higher CCI values indicate a more compact chromatin structure. **c** Transcript levels of *xyr1*. Transcript analysis was performed by RT-qPCR using *sar1* and *act* genes for normalization. Results are given as relative transcript ratios and refer to a reference sample (QM6a, NC). All values are the means of results from three independent experiments. Error bars indicate standard deviations

**Fig. 3:**
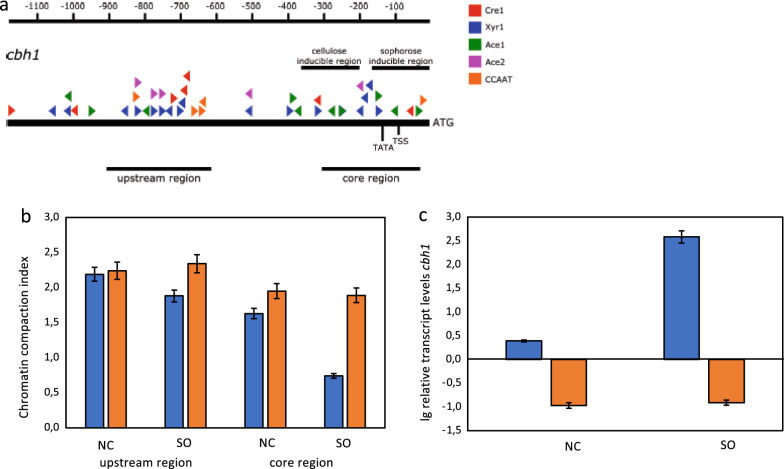
Analysis of the chromatin status upstream of the *cbh1* gene and its transcript formation. **a** Schematic drawing of the upstream region of the *cbh1* gene. The black bars indicate the regions chosen for the CHART-PCR. Triangles indicate putative binding sites for transcription factors (colour-code provided). TATA indicates the position of a TATA-like box. TSS indicates the position of the putative transcription start site. Numbers on top indicate distance from ATG. *T. reesei* Iogen-M10 (blue bars) and Iogen-M10 (cel −) (orange bars) strains were pre-grown and transferred to sophorose (SO) or medium without carbon source (NC) and incubated for 3 h. **b** CHART-PCR analysis of the two chosen regions. The chromatin compaction index (CCI) indicates the accessibility of DNA to DNase I digestion using normalization to *sar1* and *act* upstream regions. Higher CCI values indicate a more compact chromatin structure. **c** Transcript levels of *cbh1*. Transcript analysis was performed by RT-qPCR using *sar1* and *act* genes for normalization. Results are given as relative transcript ratios in logarithmic scale and refer to a reference sample (QM6a, NC). All values are the means of results from three independent experiments. Error bars indicate standard deviations

**Fig. 4:**
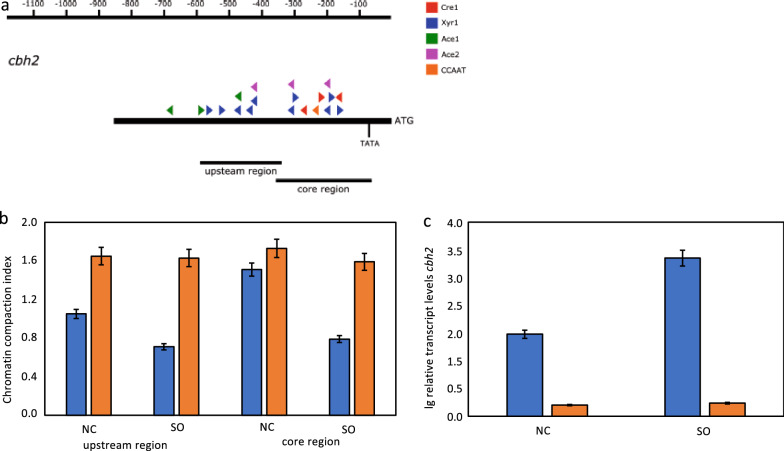
Analysis of the chromatin status upstream of the *cbh2* gene and its transcript formation. **a** Schematic drawing of the upstream region of the *cbh2* gene. The black bars indicate the regions chosen for the CHART-PCR. Triangles indicate putative binding sites for transcription factors (colour-code provided). TATA indicates the position of a TATA-like box. Numbers on top indicate distance from ATG. *T. reesei* Iogen-M10 (blue bars) and Iogen-M10 (cel −) (orange bars) strains were pre-grown and transferred to sophorose (SO) or medium without carbon source (NC) and incubated for 3 h. **b** CHART-PCR analysis of the two chosen regions. The chromatin compaction index (CCI) indicates the accessibility of DNA to DNase I digestion using normalization to *sar1* and *act* upstream regions. Higher CCI values indicate a more compact chromatin structure. **c** Transcript levels of *cbh2*. Transcript analysis was performed by RT-qPCR using *sar1* and *act* genes for normalization. Results are given as relative transcript ratios in logarithmic scale and refer to a reference sample (QM6a, NC). All values are the means of results from three independent experiments. Error bars indicate standard deviations

In order to test the above-mentioned speculation, we decided to use chromatin accessibility real-time PCR (CHART-PCR) assays to investigate the chromatin status of upstream regions of genes relevant for cellulase production. Due to its essential role for cellulase formation, we started with the analysis of three upstream regions of *xyr1*. One region was chosen as it might be a core region for transcription initiation (bearing a TATA-box-like element), one region was chosen due to a high number of regulatory *cis* elements, and a third region in between, which does not comprise any known element that is putatively relevant for cellulase formation, was included as well (Fig. 2a). Iogen-M10 and Iogen-M10 (cel −) were pre-grown and transferred to a condition inducing cellulase production (i.e., sophorose) and to a medium without any carbon source and were incubated for 3 h. Chromatin compaction was found in the core region and the region bearing the regulatory *cis* elements in the (cel −) strain under both conditions, while the chromatin status of the middle region was similar in both strains under both conditions (Fig. 2b). To see whether the chromatin compaction that was observed in two regions in the (cel −) strain does affect transcript formation, we determined *xyr1* transcript levels by RT-qPCR. While no substantial change in *xyr1* transcript levels between Iogen-M10 and the (cel −) strain was found in case of the control condition (without any carbon source), under inducing conditions the *xyr1* transcript level was clearly lower in the (cel −) strain (Fig. 2c). The same experimental design was applied for promoter regions of the *cbh1* and *cbh2* genes. In both cases, two regions, a potential core region and a region further upstream which is strongly enriched in *cis* elements putatively relevant for cellulase expression, were investigated (Figs. 3a, 4a). For *chb1*, chromatin compaction was detected in the (cel −) strain in the core region under both conditions and in the upstream region under inducing conditions (Fig. 3b). A strong decrease in *cbh1* transcript was found for the (cel −) strain under both conditions compared to Iogen-M10 (Fig. 3c). For *cbh2*, chromatin compaction was detected in the (cel −) strain in both regions under both conditions (Fig. 4b). Transcript levels of *cbh2* were strongly reduced in the (cel −) strain compared to Iogen-M10 under both conditions (Fig. 4c). Summarizing, the (cel −) strain has a more compact chromatin in promoter regions of genes responsible for cellulase production, which is reflected in strongly reduced transcript levels of these genes. The latter is in accordance with the observed loss of cellulase production in the (cel −) strain and suggests a relation of this phenotype to epigenetic mechanisms.

### A method for induced strain degeneration

**Fig. 5:**
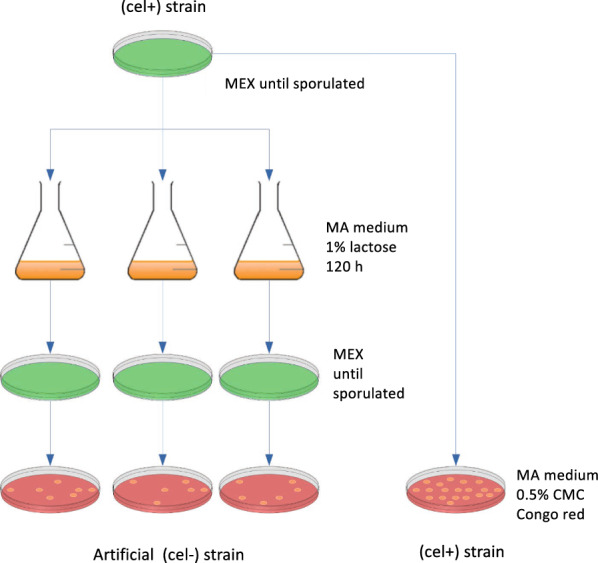
Schematic presentation of the workflow for induced strain degeneration. A productive strain (cel  +) is cultivated on MEX plates until sporulation. The spores are used to inoculate liquid MA medium containing 1% (w/v) lactose in triplicates. Mycelium is harvested after 120 h of cultivation and spread on MEX plates for sporulation. The obtained spores and spores from the initially used (cel  +) strain are spread on plates containing MA medium with 0.5% (w/v) CMC and incubated for 72 h. The plates are stained with Congo Red. The number of colonies without a halo, i.e., (cel −) colonies, can be plotted as the percentage of the total number of colonies

**Fig. 6:**
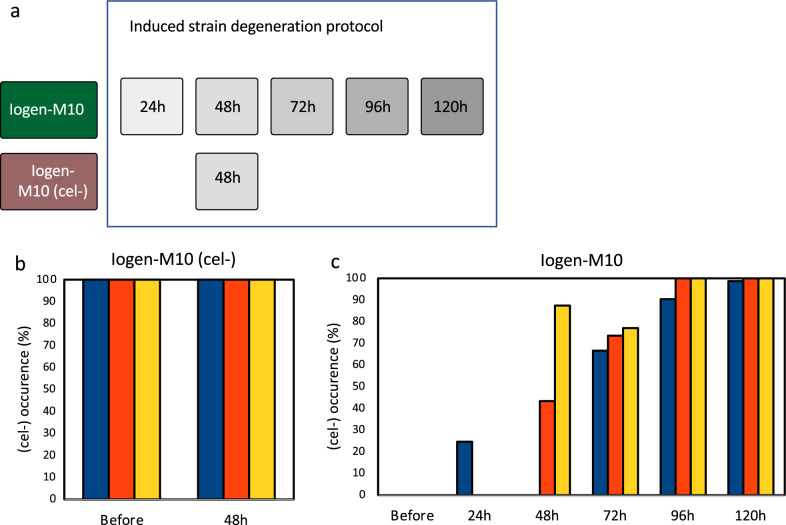
Phenotypic characterization of artificially generated (cel −) strains. **a** *T. reesei* Iogen-M10 and Iogen-M10 (cel −) strains were employed in the protocol for ISD in biological triplicates and samples were taken after the indicated cultivation times. The occurrence of the (cel −) phenotype in the two investigated strains Iogen-M10 (cel −) (as a control) (**b**) and Iogen-M10 (**c**) before their employment in the protocol for ISD (“Before”) and after the indicated cultivation time is given in % of the whole population. The three bars represent the biological triplicates. If no bar is depicted, no (cel −) colonies were observed

As mentioned above, observations indicated that different strains have a differently strong tendency to degenerate. Besides, it is obvious that any strategies for the preventing or reversing the degeneration phenomenon need a lab-scale format for a fast and standardized monitoring of the success of such strategies. For this purpose, a method was developed that allows the controlled trigger of strain degeneration and once the workflow is completed, it yields a degeneration rate, i.e., the percentage of (cel −) colonies within a whole population. Figure 5 displays the main workflow of the developed protocol for induced strain degeneration (ISD). Briefly, spores of the strain to be tested are generated on full medium. Harvested spores are split. One part is used to inoculate liquid minimal medium containing lactose as carbon source and perform an extended cultivation. Mycelium is harvested and again brought to sporulation on full medium. The obtained spores and the other part of the initial spores are used in a cellulase activity assay on plates, i.e., growth on minimal medium containing carboxymethyl cellulose as the only carbon source. This is followed by staining using Congo red for a clear visualization of a clearing zone (halo) around the colonies, which develops as the result of cellulolytic activity. The colonies with a halo are counted and related to the total number of colonies. For the development of the ISD protocol the production strain Iogen-M10 was used and samples were analysed every 24 h during the extended cultivation (Fig. 6a) and compared with the initial spores that were not submitted to the ISD protocol. As a control the strain, Iogen-M10 (cel −) was applied (Fig. 6a). This strain was isolated from a production process during which the strain degeneration phenomenon was observed. Figure 6 displays the occurrence of the (cel −) population, which was already at 100% in the (cel −) control strain (Fig. 6b) and developed over time in the producing strain until it also reached a uniform, non-producing population at the later stages of the ISD (96 h, 120 h) (Fig. 6c).

### The extent of degeneration relates to the productivity of a strain

**Fig. 7:**
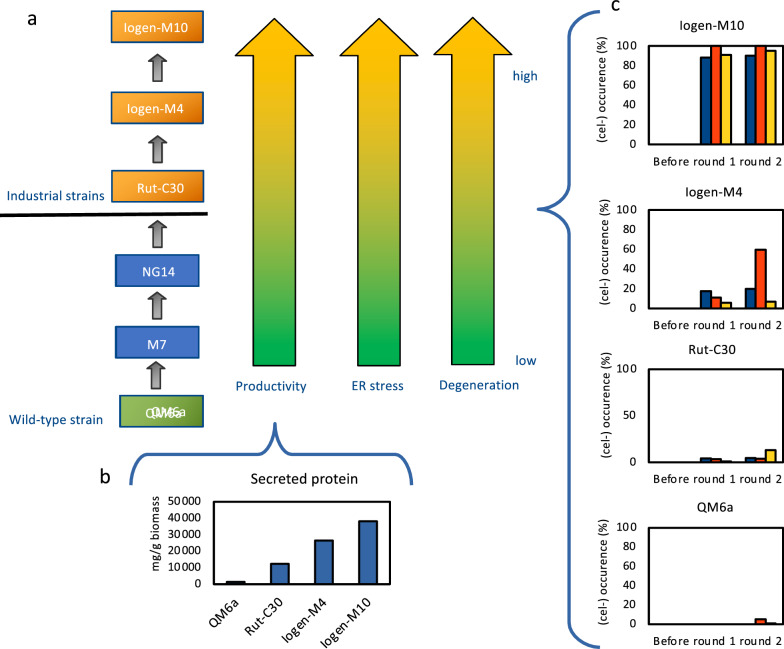
Determination of the degeneration behaviour and the productivity of *T. reesei* strains. **a** The *T. reesei* strain lineage is displayed from the wild-type strain (QM6a) over strains M7 and NG14 in three rounds of mutagenesis to Rut-C30, which is the first strain involved in industrial purposes and the last in the lineage publicly available. Strains Iogen-M4 and Iogen-M10 were further developed from Rut-C30. **b** Secreted protein is given for QM6a, Rut-C30, Iogen-M4 and Iogen-M10 in mg/g biomass. **c** After submitting the four strains to the ISD protocol in two consecutive rounds the degeneration rate was determined and is given in % of the whole population. The three bars represent the biological triplicates. If no bar is depicted, no (cel −) colonies were observed

Since a protocol for standardized determination of the degeneration behaviour of a certain strain was now available, we decided to compare differently strongly evolved strains with regard to cellulase production. In this experiment we used the wild-type strain QM6a, strain Rut-C30, which was developed from QM6a by three rounds of mutagenesis and screening for increased protein secretion, Iogen-M4, which is a moderately producing industrial strain in the same strain lineage, and Iogen-M10, the before investigated, highly producing industrial strain, which also belongs to the same strain lineage (Fig. 7a). First, the total secreted protein was determined as an indicator for the productivity of the strains (Fig. 7b). The obtained results reflect the order of development of these strains for increased protein production. Second, the four strains were applied in the ISD protocol and degeneration rates were determined (Fig. 7c). While no degeneration was observed in case of the wild-type QM6a, a small (cel −) population developed in case of Rut-C30, an average degeneration rate of 10% was determined in case of Iogen-M4, and a rate close to 100% was found again in case of Iogen-M10 (Fig. 7c). Since no degeneration was observed for the wild-type strain at all, colonies were subjected to a second round of the ISD protocol to test whether the repeated exposure to cellulase-inducing conditions might more strongly trigger any degeneration. Thus, spores of all four strains after the first round of the ISD protocol were subjected to it a second time. Indeed, in case of the wild-type QM6a now low numbers of non-producing colonies were found, and in the case of Rut-C30 and Iogen-M4 a bigger (cel −) population developed. In the case of Iogen-M10 the (cel −) population was again close to 100% (Fig. 7c).

In relation to this finding, we also tested whether the presence of DTT changes the degeneration behaviour. For this purpose, we only used the hard-to-degenerate wild-type strain QM6a, again in two rounds of the ISD protocol with addition of DTT after 72 h of incubation. We observed the formation of a higher number of (cel −) colonies after the second round of the ISD in the presence of DTT (in average 11%) compared to the absence of DTT in the earlier experiment (Fig. 7c). This finding together with the fact that higher productive strains have a stronger tendency to degenerate than moderately productive strains suggests a relation between degeneration phenomenon, productivity and ER stress (Fig. 7c).

### The development towards a (cel −) population is not driven by sensing any compounds

**Fig. 8:**
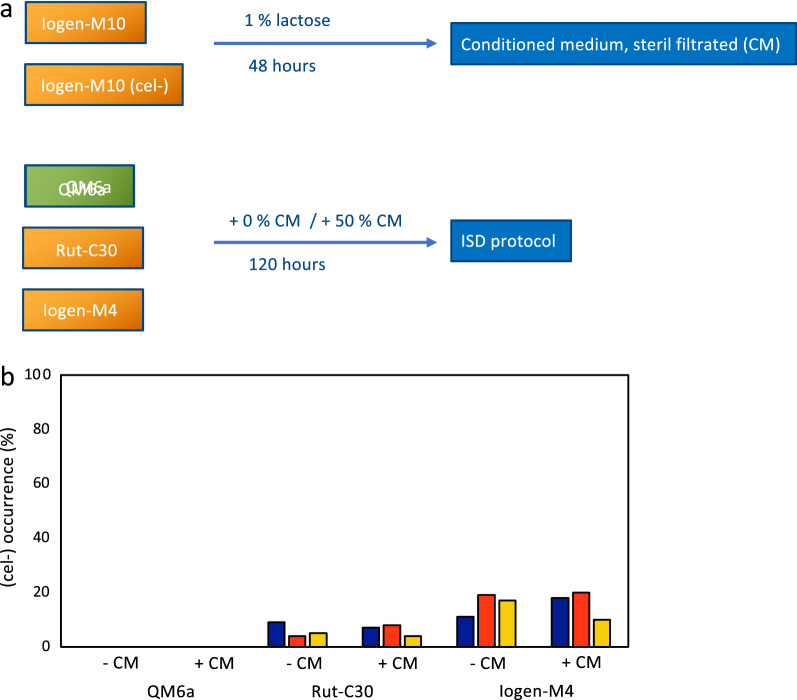
Determination of the degeneration rate after growth in conditioned medium. **a** Schematic presentation of the experimental workflow. *T. reesei* Iogen-M10 and Iogen-M10 (cel −) strains were grown on 1% lactose for 48 h. Supernatants were sterile filtered and used to mix with fresh medium in the indicated ratio. *T. reesei* QM6a, Rut-C30 and Iogen-M4 strains were submitted to the ISD protocol under standard condition and using the mixed medium. **b** The degeneration rate was determined and is given in % of the whole population. The three bars represent the biological triplicates. If no bar is depicted, no (cel −) colonies were observed

We earlier in this manuscript reported that a (semi-cel −) population irreversibly develops towards a full (cel −) population. This prompted us to test whether either a highly productive or a degenerated strain might release any compound(s) that can be sensed by the productive cell and triggers mechanisms to turn into a non-producing cell. To investigate this question, we used supernatants of cultivations of Iogen-M10 and its (cel −) counterpart to mix these conditioned media with fresh medium. For a detailed presentation of the experimental design, please refer to Fig. 8a. Strains that have different degeneration rates, namely QM6a, Rut-C30 and Iogen-M4, were submitted to the ISD protocol under standard conditions as control and using the mixed medium to test the possibility. Interestingly, for none of the strains a changed degeneration rate was found (Fig. 8b) indicating that it is not the presence of any compound that triggers the occurrence of the (cel −) population.

## Discussion

Strain degradation is an often-observed phenomenon in eukaryotic microorganisms and spans from the loss of virulence of pathogenic microorganisms while studying them in the lab to the loss of productivity of industry strains used in biotechnology. Nonetheless, this phenomenon hampers basic research approaches as well as biotechnological processes. There are no reports about studies towards the understanding of the underlying mechanisms nor the prevention of this phenomenon.

One possible explanation for the occurrence of degeneration could be genomic imprinting. In this study, we found a relation between the degeneration rate of a strain and its productivity. Definitely, elevated stress caused for example by high levels of protein secretion, could be a crucial factor for the loss of productivity and the development of the (cel −) phenotype. Notably, it has been established in higher eukaryotes, as animals and plants, that epigenetic regulation and stress responses are closely interlinked (e.g., [21–23]). During the last two decades, quite some evidence was provided that cellulase expression in *T. reesei* is subject to epigenetic regulation through chromatin remodelling and/or DNA methylation. For example, the investigation of nucleosome positioning on the *cbh2* promoter of wild-type-like strains showed a dependence on the carbon source, i.e., cellulase-inducing versus repressing conditions [24]. This study provided indication that strain Rut-C30 loses a clear nucleosome positioning on the *cbh2* promoter independent of the carbon source used for cultivation. This led to the speculation that in Rut-C30 at least some parts of the genome (possibly including cellulase-encoding genes) are packed in a less condensed chromatin structure compared to other more wild-type-like strains. Later, Rassinger and co-workers reported an observation that reinforces this presumption: the *cbh1* promoter of Rut-C30 cultivated on lactose has a stable open chromatin status while transcript levels are rising [25]. Our study suggests that the advantage of open chromatin in promoters of genes relevant for cellulase production, which was gained in this strain lineage, is lost in the (cel −) strains.

Another link between cellulase gene expression and chromatin structure was demonstrated in *T. reesei* strains possessing a deletion of the *lae1* gene. Lae1 is the ortholog to *laeA* of *Aspergillus* spp., which recently was identified as a regulator of secondary metabolism [26] by acting as histone methyltransferase in chromatin remodelling (e.g., [27]). Deletion of Lae1 in *T. reesei* led to a loss of cellulase production, pointing to an influence of chromatin remodelling on the regulation of the cellulase expression [28].

Certainly, it remains the possibility that the (cel −) phenotype develops due to the occurrence and build-up of a genetic mutation. For example, early efforts for strain improvement by random mutagenesis did not only yield successful lineages as the one towards Rut-C30. Also strains without the ability to grow on cellulose or from cellulases even under inducing conditions were obtained (e.g., [29]). To identify possible genetic reasons for this phenotype, the whole genome of one of such strains, i.e., QM9136, was sequenced. For this strain, only a low number of mutations could be detected, however, one of them leading to a frameshift in *xyr1*, which is highly likely the cause of the cellulase negative phenotype [30]. The possibility that a mutation in the *xyr1* structural gene or upstream region might be responsible for the here described (cel −) phenotype was ruled out. Besides, it needs to be considered that the *xyr1* frame shift in QM9136 led to an immediate loss of cellulase formation, while in case of the strain degeneration phenomenon, the (cel −) population gradually develops. Anyhow, only whole genome sequencing of a number of strains in the investigated Rut-C30 lineage and their respective (cel −) counterparts would allow to assess the impact of genetic mutations on the development of a degenerated population.

Altogether, only the understanding of the mechanism(s) causing the strain degeneration will give the possibility to prevent the undesired phenomenon. As discussed above several regulatory levels that contribute to gene expression cannot be ruled out at the moment and warrant for further investigations.

## Conclusions

The unpredictable development of a non-producing population of industrially used *T. reesei* strains during cultivation for cellulase production is a not understood phenomenon. The development of a protocol for ISD revealed that the rate of the degeneration correlates with the productivity of strains. Once turned into a (cel −) strain, the phenotype cannot be reversed, and (semi-cel −) strains gradually develop into the full (cel −) population suggesting the involvement of epigenetic mechanism. The chromatin of promoter regions of genes relevant for cellulase production become more condensed in (cel −) strains and transcript levels of the corresponding genes are strongly reduced. The impact of genetic mutations cannot be fully ruled out yet, however, mutations leading to a deficient Xyr1 can be excluded.

## Methods

### Strains and cultivation conditions

*T. reesei* Iogen-M4, Iogen-M4 (cel −) and Iogen-M10 and Iogen-M10 (cel −) strains (proprietary of Novozymes, Bagsvaerd, Denmark) were maintained on potato dextrose agar (PDA) plates or on plates containing 3% (w/v) malt extract, 0.1% (w/v) peptone and 1.5% (w/v) agar (MEX plates). For storage, the strains were kept on plates at 4 °C (short-term) or as spore suspensions in 25% glycerol at − 80 °C (long-term).

Cultivation of *T. reesei* for enzyme production was performed in a 14-L pilot-scale bioreactor (Model MF114 New Brunswick, Eppendorf, Hamburg, Germany). For the inoculum, spores from a single PDA plate were transferred into 750 mL of liquid Berkley medium (pH 5.5) [10.40 g/L (NH_4_)_2_SO_4_, 2.00 g/L KH_2_PO_4_, 0.31 g/L MgSO_4_·7H_2_O, 0.53 g/L CaCl_2_·2H_2_O, 1 mL/L trace element solution (5 g/L FeSO_4_·7H_2_O, 1.6 g/L MnSO_4_·H_2_O, 1.4 g/L ZnSO_4_·7H_2_O)] supplemented with 5.1 g/L corn steep liquor powder and 10 g/L glucose. The concentration of the inoculate for all cultivations or fermentations was 10^6^ spores/mL. Incubation was followed at 28 °C for 3 days at 100 rpm. The whole content was used as inoculum of 10-L Initial Pilot Medium (pH 5.5) [2.20 g/L (NH_4_)_2_SO_4_, 1.39 g/L KH_2_PO_4_, 0.70 g/L MgSO_4_·7H_2_O, 0.185 g/L CaCl_2_·2H_2_O, 6.00 g/L corn steep liquor powder, 13 g/L glucose, 0.38 mL/L trace element solution (5 g/L FeSO_4_·7H_2_O, 1.6 g/L MnSO_4_·H_2_O, 1.4 g/L ZnSO_4_·7H_2_O)]. First, batch mode was used until the glucose was depleted, then, feeding with a solution containing cellulase-inducing carbohydrate (proprietary of Novozymes, Bagsvaerd, Denmark) was started at a carbon addition rate of 0.4 g carbon per L per h. Cultivation was performed at 28 °C, an agitation rate of 500 rpm, and air sparging at 8 standard L per min for 168 h. The pH was maintained at 4.0–4.5 during the batch mode and at pH 3.5 during fed-batch using 10% (w/v) NaOH solution. Samples of 100 mL culture broth were taken every 24 h and used for determination of biomass, protein content and cellulase activity.

Cultivation of *T. reesei* for ISD was performed in 20 mL of Mandels–Andreotti (MA) medium containing 1% (w/v) lactose and 0.1% (w/v) peptone. After determining the spore count under a light microscope, 2 × 10^7^ spores were used for inoculation and incubation of the cultures was followed at 30 °C and 160 rpm for 120 h. For the advanced degeneration, 20 mM dithiothreitol (DTT) was added after 72 h and incubation was followed for 120 h in total. Cultures were harvested, regenerated on MEX plates and spores were eventually applied in a second round of ISD or directly used for determination of cellulase activity.

### Biomass determination

For the gravimetric determination of the biomass content in samples from cultivation in a bioreactor, the constant weight of culture broth sample volumes of 5–10 mL was measured. Afterwards they were filtered using vacuum through glass fibre filters and oven-dried at 100 °C for 12 h.

### Protein content determination

The protein concentration in supernatants of the culture broth was determined by Bradford reagent followed by spectrophotometric measurement at 595 nm. For quantification, a standard curve using a cellulase mixture of known composition was employed.

The specific productivity (*q*_*p*_) gives mg protein produced per g of biomass per hour of cultivation.

### Cellulase activity assay

To determine whether or not the samples from cultivation in a bioreactor have cellulase activity, biomass samples were plated on PDA and incubated at 30 °C for 5 days until sporulation. In case of the investigation of the effects of Trichostatin A, 10 µM were added to the plates. Spores were harvested and about 50–100 spores were spread on plates containing minimal medium and 1% (w/v) acid-swollen cellulose (ASC) and 0.7% ox gall. These plates were incubated at 30 °C for 6 days and afterwards at 50 °C for 20 h. The formation of a clearing zone around a colony indicates cellulase secretion and activity. Cellulase producing and non-producing colonies were counted and the (cel −) occurrence was given as the percentage of non-producing colonies from the total number of colonies.

For determination of cellulase activity in samples from the ISD protocol, biomass samples were plated on MEX plates and incubated 30 °C for 5 days until sporulation. Spores were harvested and about 100–200 spores were spread on plates containing MA medium supplemented with 0.5% (w/v) CMC (carboxymethylcellulose sodium salt, high viscosity), 0.1% (w/v) peptone and 2.0% (w/v) agar. These plates were incubated at 30 °C for 3 days and afterwards at 50 °C for 4 h. Plates were stained with a 10-mL aliquot of Congo red dye (2.5 g/L) for 15 min at room temperature with gentle stirring, followed by washing the plates with 10 mL of 1 M NaCl. The formation of a clearing zone around a colony indicates cellulase secretion and activity. Cellulase producing and non-producing colonies were counted and the (cel −) occurrence was given as the percentage of non-producing colonies from the total number of colonies.

### Biolog microarray technique

The global carbon assimilation profiles were evaluated by using Biolog FF MicroPlate (Biolog, Inc., Hayward, CA, USA) following a previously described protocol [31], with minor modifications as follows: the inoculum was prepared from cultures on PDA plates incubated at 30 °C. Mycelial growth was measured after 18, 24, 30, 36, 42, 48, 66, 72, and 90 h using biological triplicates.

### Sequencing of the *xyr1* locus

The structural gene of *xyr1* and approximately 900 bp upstream of the coding region were amplified as three overlapping fragments from 1200 to 1500 bp using the Q5^®^ High-Fidelity DNA Polymerase (New England Biolabs, Ipswich, MA, USA). Primer sequences are provided in Table 1. The amplicons were purified from an agarose gel using the GeneJET Gel Extraction Kit (Thermo Fisher Scientific, Waltham, MA, USA) and cloned using the CloneJET PCR Cloning Kit (Thermo Fisher Scientific, Waltham, MA, USA). Fifty µL of TOP10 competent *E. coli* cells were transformed with the ligation mixes and plated on LB agar supplemented with ampicillin. Resulting single colonies were inoculated overnight in 5 mL of liquid LB medium supplemented with ampicillin and prepped using the GeneJET Plasmid Miniprep Kit (Thermo Fisher Scientific, Waltham, MA, USA). The plasmids were sent to Microsynth AG (Balgach, Switzerland) for an analysis by Sanger sequencing. All kits and reagents were used according to the manufacturers’ instructions.

**Table 1:** Oligonucleotides used in this study

Name	Sequence (5′–3′)	Employment
PCR1_fwd	TCCATCCCCATCCCGTTCTCCATCCATCCATG (32)	Sequencing of *xyr1* locus
PCR1_rev	CTAAACAAGATCGATCAGTACATG (24)	
PCR2_fwd	ATTCAACGGGTACTGCTGGG (20)	
PCR2_rev	GTTCAAGTCGTGCTCATCCAC (21)	
PCR3_fwd	CCACCTGCCAACCAGGAGG (19)	
PCR3_rev	TAGAGGGCCAGACCGGTTC (19)	
epiactinTr_f	CTTCCCTCCTTTCCTCCCCCTCCAC	CHART-PCR
epiactinTr_r	GCGACAGGTGCACGTACCCTCCATT	
epicbh1_1Tr_f	AAGGGAAACCACCGATAGCAGTGTC	
epicbh1_1Tr_r	TTTCACTTCACCGGAACAAACAAGC	
epicbh1_2Tr_f	GGATCGAACACACTGCTGCCTTTAC	
epicbh1_2Tr_r	GGTTTCTGTGCCTCAAAAGATGGTG	
epicbh2_1Tr_f	CGGATCTAGGGCAGACTGGGCATTG	
epicbh2_1Tr_r	GTGTAGTGTTGCGCTGCACCCTGAG	
epicbh2_2Tr_f	TGCAGCGCAACACTACACGCAACAT	
epicbh2_2Tr_r	TGCGCCTCATACAGGGTCACAGTCC	
episar1Tr_f	GTCAGGAAATGCCGCACAAGCAAGA	
episar1Tr_r	TGTGTTTTACCGCCTTGGCCTTTGG	
epixyr1_1Tr_f	CCTTTGGCCATCTACACAAGAGCAA	
epixyr1_1Tr_r	CGCAATTTTTATTGCTGTTCGCTTC	
epixyr1_2Tr_f	CCGACAGCAGCAGTAGTCAGGTTTT	
epixyr1_2Tr_r	TAGGCAGAATAGCGACGGAGAGGAT	
epixyr1_3Tr_f	GGCAGCCGTGTAGCTTGTCA	
epixyr1_3Tr_r	GGAATCAAACCGTCGCCTCTT	
act_fw	TGAGAGCGGTGGTATCCACG	qPCR
act_rev	GGTACCACCAGACATGACAATGTTG	
cbh1f	GATGATGACTACGCCAACATGCTG	
cbh1r	ACGGCACCGGGTGTGG	
cbh2f	CTATGCCGGACAGTTTGTGGTG	
cbh2r	GTCAGGCTCAATAACCAGGAGG	
sar1fw	TGGATCGTCAACTGGTTCTACGA	
sar1rev	GCATGTGTAGCAACGTGGTCTTT	
xyr1f	CCCATTCGGCGGAGGATCAG	
xyr1r	CGAATTCTATACAATGGGCACATGGG	

### CHART-PCR

DNase I digestion of chromatin and DNA extraction were carried out as described by [32] with minor modifications. Mycelia were harvested by filtration, pressed dry with filter paper, frozen in liquid nitrogen, and ground to a fine powder. Portions (100 mg) of the powder were suspended in 1-mL aliquots of nuclease digestion buffer (250 mM sucrose, 60 mM KCl, 15 mM NaCl, 0.05 mM CaCl_2_, 3 mM MgCl_2_, 0.5 mM DTT, 15 mM Tris–HCl pH 7.5), and 100-µL samples of the digestion mixture were incubated with 10 U of RQ1 RNase-free DNase I (Promega, Madison, WI, USA) for 2.5 min at 37 °C. The reaction was stopped by adding 100 µL of 40 mM EDTA, 2% SDS, followed by two rounds of phenol–chloroform extraction and one round of chloroform extraction. Samples were then treated with 10 μg/mL of RNase A for 15 min at 37 °C and precipitated with ethanol. DNA pellets were suspended in 100 µl of 5 mM Tris–HCl pH 7.5. A control without DNase I was included to monitor endonuclease activity. qPCR analysis of the DNase I-treated samples was performed to measure the relative abundance of target regions. PCRs were performed in a Rotor-Gene Q system (Qiagen, Hilden, Germany). All reactions were performed in triplicate. The amplification mixture (final volume 20 µL) contained 10 µL of 2  ×  iQ SYBR Green Mix (Bio-Rad, Hercules, CA, USA), 200 nM forward and reverse primers and 10 ng of DNA. Primer sequences are provided in Table 1. Cycling conditions were as follows: 3 min initial denaturation at 95 °C, followed by 40 cycles of 15 s at 95 °C and 60 s at 60 °C. The amount of intact input DNA of each sample was calculated by comparing the threshold values of the PCR amplification plots with a standard curve generated for each primer set using serial dilutions of genomic, undigested DNA. The chromatin compaction index (CCI) was defined as:

$$\mathrm{CCI}= \frac{\mathrm{D}s}{(\mathrm{D}c1+\mathrm{D}c2)/2},$$

where D*s* is the amount of intact DNA detected for a target region and D*c*1 and D*c*2 are the amounts of intact DNA detected for the promoter regions of *sar1* and *act*, respectively, used as reference genes for normalization.

### Transcript analysis

Fungal mycelia were homogenized in 1 mL of peqGOLDTriFast DNA/RNA/protein purification system reagent (PEQLAB VWR, Radnor, Pennsylvania, USA) using a FastPrep(R)-24 cell disrupter (MP Biomedicals, Santa Ana, CA, USA). RNA was isolated according to the manufacturer’s instructions, and the concentration was measured using the NanoDrop 1000 (Thermo Fisher Scientific, Waltham, MA, USA). Reverse transcription of the isolated mRNA was carried out using the RevertAid™ H Minus First Strand cDNA Synthesis Kit (Thermo Fisher Scientific, Waltham, MA, USA) according to the manufacturer’s instructions.

Quantitative PCR (qPCR) was performed in a Rotor-Gene Q system (Qiagen, Hilden, Germany). Reactions were performed in technical duplicates or triplicates. The amplification mixture (final volume of 15 μL) contained 7.5 μL of 2  ×  iQ SYBR Green Mix (Bio-Rad, Hercules, California, USA), 100 nM forward and reverse primer, and 2.5 μL of cDNA (diluted 1:20). Primer sequences are provided in Table 1. Data normalization using *sar1* and *act* as reference genes and calculations were performed as previously published [33].

## Data Availability

All data generated or analysed during this study are included in this published article. *Trichoderma reesei* strains Iogen-M4 and Iogen-M10 are Novozymes’ proprietary strains.

## Abbreviations

ASC: Acid-swollen cellulose
CCI: Chromatin compaction index
CHART-PCR: Chromatin accessibility real-time PCR
CM: Conditioned medium
CMC: Carboxymethyl cellulose
DTT: Dithiothreitol
ER: Endoplasmic reticulum
ISD: Induced strain degeneration
LB: Lysogeny broth
MA: Mandels–Andreotti
MEX: Malt extract
NC: No carbon source
PDA: Potato dextrose agar
qPCR: Quantitative PCR
RT-qPCR: Reverse transcription quantitative PCR
SO: Sophorose
